# From friends to foes: fungi could be emerging marine sponge pathogens under global change scenarios

**DOI:** 10.3389/fmicb.2023.1213340

**Published:** 2023-08-15

**Authors:** Yordanis Pérez-Llano, Luis Andrés Yarzábal Rodríguez, Esperanza Martínez-Romero, Alan D. W. Dobson, Nina Gunde-Cimerman, Vitor Vasconcelos, Ramón Alberto Batista-García

**Affiliations:** ^1^Centro de Investigación en Dinámica Celular, Instituto de Investigación en Ciencias Básicas y Aplicadas, Universidad Autónoma del Estado de Morelos, Cuernavaca, Morelos, Mexico; ^2^Center for Genomic Sciences, Autonomous National University of Mexico (UNAM), Cuernavaca, Morelos, Mexico; ^3^Carrera de Bioquímica y Farmacia, Unidad de Salud y Bienestar, Universidad Católica de Cuenca, Cuenca, Ecuador; ^4^School of Microbiology, University College Cork, Cork, Ireland; ^5^Department of Biology, Biotechnical Faculty. University of Ljubljana, Ljubljana, Slovenia; ^6^CIIMAR – Interdisciplinary Centre of Marine and Environmental Research, University of Porto, Matosinhos, Portugal; ^7^Faculty of Sciences, University of Porto, Porto, Portugal

**Keywords:** sponge microbiome, sponge holobiont, fungal-based probiotics, sponge-derived fungi, symbiosis continuum

## Abstract

Global change, experienced in the form of ocean warming and pollution by man-made goods and xenobiotics, is rapidly affecting reef ecosystems and could have devastating consequences for marine ecology. Due to their critical role in regulating marine food webs and trophic connections, sponges are an essential model for studying and forecasting the impact of global change on reef ecosystems. Microbes are regarded as major contributors to the health and survival of sponges in marine environments. While most culture-independent studies on sponge microbiome composition to date have focused on prokaryotic diversity, the importance of fungi in holobiont behavior has been largely overlooked. Studies focusing on the biology of sponge fungi are uncommon. Thus, our current understanding is quite limited regarding the interactions and “crosstalk” between sponges and their associated fungi. Anthropogenic activities and climate change may reveal sponge-associated fungi as novel emerging pathogens. Global change scenarios could trigger the expression of fungal virulence genes and unearth new opportunistic pathogens, posing a risk to the health of sponges and severely damaging reef ecosystems. Although ambitious, this hypothesis has not yet been proven. Here we also postulate as a pioneering hypothesis that manipulating sponge-associated fungal communities may be a new strategy to cope with the threats posed to sponge health by pathogens and pollutants. Additionally, we anticipate that sponge-derived fungi might be used as novel sponge health promoters and beneficial members of the resident sponge microbiome in order to increase the sponge's resistance to opportunistic fungal infections under a scenario of global change.

## Introduction

Sponges are early-derived and simple metazoans that have significant functions in marine ecosystems, including sediment stabilization, nutrient cycling, and the provision of habitats for numerous species (van Soest et al., 2012; Zhang et al., 2019). The incredible global diversity of sponges, surpassing 18,000 recognized species, spans a vast range of environments. The presence of sponges in all types of waters, from fresh to saline, from intertidal to deep-sea, from tropical to occasionally frozen aquatic systems, clearly demonstrates their capacity to respond and adapt to a broad range of environmental conditions (van Soest et al., 2012). Nonetheless, sponges and coral reef systems are currently exposed to ocean warming, acidification, and pollution by xenobiotics, likely to have devastating consequences for marine ecosystems (Carballo and Bell, 2017). Recurrent epidemic disease outbreaks pose a significant challenge for sponge populations both for long-lived slow-growing species, and for biotechnologically important species farmed in aquaculture systems (Blanquer et al., 2016; Belikov et al., 2019). Thus, both local and global pressures on sponge ecosystems are leading to an increasingly deteriorating health of sponges across the world (Webster, 2007; Blanquer et al., 2016; Pita et al., 2018; Belikov et al., 2019; Taylor et al., 2021).

Microbes are regarded as major contributors to the health and survival of sponges (Carballo and Bell, 2017). The diversity of bacteria and to a lesser extent of archaea, associated with sponges has been extensively investigated (Alex et al., 2013; Alex and Antunes, 2015; Thomas et al., 2016; Glasl et al., 2018; Turon et al., 2019; Vargas et al., 2021). Strikingly, except for a few published studies (Nguyen and Thomas, 2018; Hardoim et al., 2021), the eukaryotic fraction of this sponge microbiome is almost unknown (Pita et al., 2018). This is surprising as fungi promote health, defense, nutrition, and survival in their ecological interaction with plant and animal hosts and might perform similar services for sponges. On the other hand, fungi might act as *bona fide* or opportunistic pathogens for sponges as they behave similarly with other animals, including corals (Pita et al., 2018; Jones et al., 2022). Hence, in this perspective article, we propose the hypothesis that sponge-associated fungi could act as potential indicators or “forecasters” of marine environmental perturbations and even become opportunistic pathogens of sponges upon global change.

### The holobiont concept is pivotal to understanding the future ecology of marine sponges

The microbiomes of sponges are both complex and diverse, as highlighted in several studies (Thomas et al., 2016; Glasl et al., 2018; Turon et al., 2019; Vargas et al., 2021). As much as 40–60 percent of the volume of certain sponge species corresponds to abundant and diverse microorganisms that are able to colonize their mesohyl matrix (Thomas et al., 2016; Yang et al., 2019; Díez-Vives et al., 2020). It has been widely hypothesized that the nutrient flux originating from sponges benefits their resident microbial communities (Zhang et al., 2019; Hudspith et al., 2021). In turn, symbiotic microbes support their hosts by contributing to vitamin and natural product biosynthesis, promoting the production of chemical defenses, and facilitating the biodegradation of xenobiotics that reach coral reef habitats as a result of global change (Pita et al., 2018; Hudspith et al., 2021).

The collection of persistent (and usually abundant) microbial taxa that are found in all individuals of a species is regarded as the core microbiota (Astudillo-García et al., 2017). The ecological unit formed by the sponge and its core microbiota can be considered a holobiont, i.e., an entity formed by the intertwined evolution of the animal and microbial partners. The outcome of symbiosis within sponges depends, however, on the physiology of the host and individual microbes in specific environmental conditions. The lifestyles and strategies of microorganisms in their interaction with the host can fluctuate in a continuum from mutualism to parasitism as they adapt to the environmental constraints, making the holobiont and eco-evolutionary concept that is transient and plastic across spatial and temporal scales (Stengel et al., 2022).

Sponges control their microbial residents by distinguishing between foreign and symbiotic microorganisms most likely via immune-related responses (Wehrl et al., 2007). Although some progress has been made toward understanding how sponges differentiate between microbial “friends or foes” (Pita et al., 2018; Schmittmann et al., 2021), the field of sponge immunity remains at its infancy. For example, the sponge *Suberites domuncula* might recognize fungi via the β-glucans on their cell walls, but the specific effect of the cytokine produced by the activation of this signaling pathway has not been described (Perovic-Ottstadt et al., 2004). High-throughput sequencing data has revealed that sponges harbor a complex genomic repertoire of immune receptors, collectively called pattern recognition receptors (PRRs). Notably, these include Toll-like receptors (TLR), Nod-like receptors (NLR), and several members of the scavenger receptor cysteine-rich (SRCR) family; the functions of which are only beginning to be elucidated in response to microbes (Degnan, 2015).

There are conflicting reports in the literature regarding the stability and resilience of the sponge holobiont in response to global change conditions (Bell et al., 2018; Glasl et al., 2018; Posadas et al., 2022). While some studies report environmentally-induced shifts in the microbiome that precede sponge disease (Blanquer et al., 2016; Belikov et al., 2019; Turon et al., 2019; Taylor et al., 2021), others have found that sponge microbiomes can withstand sustained stress conditions (Glasl et al., 2018; Yang et al., 2019). This arises from observed changes in the species-specific dynamics of the sponge microbiomes (Yang et al., 2019), resulting in different adaptability profiles of the sponge holobionts to varying stressful conditions. Even though sponges have been predicted to potentially benefit from ocean warming and acidification (Bell et al., 2018), leading to a predominance of sponges in the benthic ecosystem in future global warming scenarios; this really only applies to a limited subset of species. The forecasted consequence is a reduction in sponge diversity in combination with an increase in microbiome diversity, resulting in reduced stability of the core microbiota (Turon et al., 2019).

In some cases, the dysbiosis of the sponge microbiome, rather than the presence of a single pathogen, has been identified as a key factor contributing to sponge disease (Glasl et al., 2018; Pita et al., 2018; Turon et al., 2019). Dysbiosis refers to the disruptions in the normal or healthy structure and function of the microbial community within the holobiont, which can have detrimental effects on the host’s health and ecological interactions. These modifications in the sponge microbiome can occur even before the host exhibits any sign of stress (Taylor et al., 2021), and have been associated with environmental pressures such as ocean warming, acidification, and exposure to pollution (Blanquer et al., 2016; Belikov et al., 2019; Botté et al., 2019; Turon et al., 2019; Taylor et al., 2021; Vargas et al., 2021). For example, after conducting electron microscopy, infection assays to propagate the syndrome in healthy sponges, and molecular community analyses, the presence of brown spot lesions and necrosis in *Ianthella basta* was attributed to environmental conditions, as these approaches failed to identify any microbial etiology. In addition, pollution affected the bacterial community of *Aplysina cauliformis* with red band syndrome (Gochfeld et al., 2012), with nutrient-enriched treatments exacerbating symptoms in this Caribbean sponge.

Abiotic factors influence not only the structure of sponge microbiomes but also their functions. For instance, CO_2_ concentration affects microbial functions in *Coelocarteria singaporensis* and *Stylissa flabelliformis* (Botté et al., 2019), which changed drastically with ocean acidification. Temperature, depth, and geographic location all affect the sponge microbial composition globally (Lurgi et al., 2019). As a result, recent studies have suggested that microbial community composition could be a predictor of sponge disease risk (Taylor et al., 2021). For example, in the marine sponge *Scopalina* sp. bacterial signatures in healthy sponges could predict disease outcomes under an ocean warming scenario.

Thus, the holobiont concept has profound implications in helping to predict the effect of future environmental variations on sponge health and benthic ecology. There is an increasing interest to understand how microbiome diversity influences the sponge immune functions under future ocean conditions (Posadas et al., 2022). This could represent a starting point to design new strategies for the conservation of sponge diversity and mitigating the global change consequences.

### From friends to foes: fungi could be emerging pathogens under global change scenarios

While most culture-independent studies on sponge microbiome composition to date have focused on prokaryotic diversity, the importance of fungi in holobiont behavior has been largely overlooked. Studies focusing on the biology of sponge fungi are uncommon compared to reports on the chemistry of natural products derived from sponge fungi (de Oliveira et al., 2020; Zhang et al., 2020).

An increasing number of fungal species have been linked to marine sponges in the last decades (Höller et al., 2000; Morrison-Gardiner, 2000; Li and Wang, 2009). In one of the most comprehensive studies of sponge-associated fungal biodiversity, Höller et al. isolated a total of 681 fungal strains from 16 sponge species from temperate, subtropical, and tropical regions (Höller et al., 2000). The isolates were distributed among 37 genera of mitosporic fungi, 13 genera of Ascomycota, and 2 genera of Zygomycota. The number of isolates per sample and the diversity of genera varied significantly between the various sites. Almost all the locations yielded *Acremonium*, *Arthrium*, *Coniothyrium*, *Fusarium*, *Mucor*, *Penicillium*, *Phoma*, *Trichoderma*, and *Verticilum* strains, though different fungal genera predominated at each site. In addition, different fungal genera predominated in different sponges sampled from the same location (Höller et al., 2000). In a different study, Morrison-Gardiner isolated 617 fungi from sediments, algae, and vertebrates/invertebrates in Australian coral reefs that included 70 Porifera sample sources (Morrison-Gardiner, 2000). The findings suggest that some reef residents might act as a natural reservoir for fungal genera that are typically connected to other organisms, whereas many of the fungi isolated from sponges were not present in other sources (Morrison-Gardiner, 2000). The main disadvantage of both studies was that the identification of fungi was achieved by morphological characterization.

A recent study based on culture-independent characterization of the sponge eukaryotic communities from seven sponge species of the Mediterranean Sea found that around 0.75% of the 18S rRNA gene reads had a fungal origin, and from these more than half could also be found in seawater (Naim et al., 2017). These led the authors to the conclusion that the presence of fungi in sponges is largely accidental and does not support the existence of a fungal community unique to sponges (Naim et al., 2017). This observation is also supported by metabarcoding studies from three co-occurring sponges from Australian benthic ecosystems that showed that, within any given sponge species, the fungal communities were found to be highly variable compared to bacterial communities (Nguyen and Thomas, 2018). In these sponges, only a few “core” fungal taxa could be identified as enriched when compared to surrounding seawater (Nguyen and Thomas, 2018). This suggests that only those fungi that can thrive in the sponge environment are selected and therefore horizontal transmission, although possible, may be uncommon in this scenario.

There is, however, observational evidence of interactions between sponges and fungi. Immunocytochemical and transmission electron microscopy evidence suggests that an endosymbiotic yeast is vertically transmitted in the marine sponge *Chondrilla* sp. (Maldonado et al., 2005). The putative horizontal gene transfer of a fungal mitochondrial intron into the genome of the sponge *Tetilla sp.* has also been considered as indirect evidence for a symbiotic relationship between fungi and a sponge (Rot et al., 2006). Koralionastes ascomycetes, found only in the ocean, are said to form their ascomata on or inside crustaceous sponges (Kohlmeyer and Volkmann-Kohlmeyer, 1990). Therefore, the symbiotic relationship between fungi and sponges is still a relatively unexplored area of biology and evolution.

Sponge pathogenic fungi, on the other hand, are also largely unexplored. In 1884, a considerable number of sponges (*Ircinia* spp.) in the Indian Ocean were affected by an unknown disease that was most probably caused by a filamentous fungus. Later, mortality between 70 and 95% of the affected individuals was recorded in commercial sponges in the Florida Keys (1939), Bahamas (1938–1939), Cuba (1939), and British Honduras (1939) (Webster, 2007). Again, unidentified filamentous fungi were observed in the tissues of the diseased sponges, but no microbiological analyses were performed to identify the etiological agent(s) (Webster, 2007; Carballo and Bell, 2017). In 2015, Sweet and coworkers described a novel disease in *Callyspongia* (*Euplacella*) aff. *Biru* sponges colonizing Maldivian ecosystems (Sweet et al., 2015). The individuals exhibited brown necrotic lesions caused by a microbial consortium, which included the fungus *Rhabdocline* sp.

While studies show that certain fungi (such as *Aspergillus sydowii*) may infect corals, there is, to the best of our knowledge, only one recent study demonstrating that a fungus (*Aspergillus tubingensis*) can cause a fatal infection in the marine sponge *Chondrosia reniformis* (Greco et al., 2019). The symptoms include loss in pigmentation, softening of the cortical portions, detachment of the outer portion of the ectosome, and, finally, death of the sponges (Greco et al., 2019). This finding takes on added significance when viewed within the framework of One Health, as *A. tubingensis* is already known to be a human pathogen (Gautier et al., 2016), raising concerns about the potential emergence of marine zoonotic diseases that could pose threats to human health in the face of a changing environment. The possibility that sponges could serve as reservoirs or substrates for the spread of fungal infections adds another layer of complexity, potentially affecting not only marine ecosystems but also terrestrial animals. Interestingly, the marine sponge *Spongia obscura* has been found to harbor *A. sydowii* without exhibiting symptoms of infection, suggesting that it could be a reservoir of this coral pathogen (Ein-Gil et al., 2009).

Fungi are known for their extremotolerance, demonstrated as the ability to endure a wide range of harsh environmental conditions. Like sponges, certain fungal species might also benefit from climate change (Jones et al., 2022). For instance, the acidification of marine waters, salinization of estuaries, and slight increases in water temperature could benefit the proliferation of many species of fungi since, as in general, they are well adapted to these conditions (Krause et al., 2013a,b). Global change could trigger the expression of genes related to toxin production, cell wall properties, osmotolerance, unicellular and dimorphic growth, motility, attachment, chemotaxis, or quorum sensing. These genes are crucial for adapting to new environments and can also contribute to virulence in pathogenic fungal ecotypes (Roik et al., 2022). The mechanisms by which fungi become harmful to sponges and the causes and triggers of the switch from non-detrimental fungal colonizer to fungal pathogen have not been explored (Webster, 2007; Greco et al., 2019).

A major obstacle in studying the ecological interactions between marine sponges and their resident microbes is the current methodology used to identify pathogens. Existing aquarium systems used in laboratory settings to replicate natural sponge microbiomes are inadequate for the safe utilization of fungal inoculants, which can pose risks to human and environmental health. For instance, the presence of *A. tubingensis*, a pathogen of sponges and an opportunistic human pathogen (Gautier et al., 2016), highlights the need for caution. Moreover traditional approaches fail to anticipate the ecological context in which interactions between sponges, resident fungi, and the changing environment may trigger previously unrecognized —but potentially lethal— infections (Greco et al., 2019).

**Figure 1:**
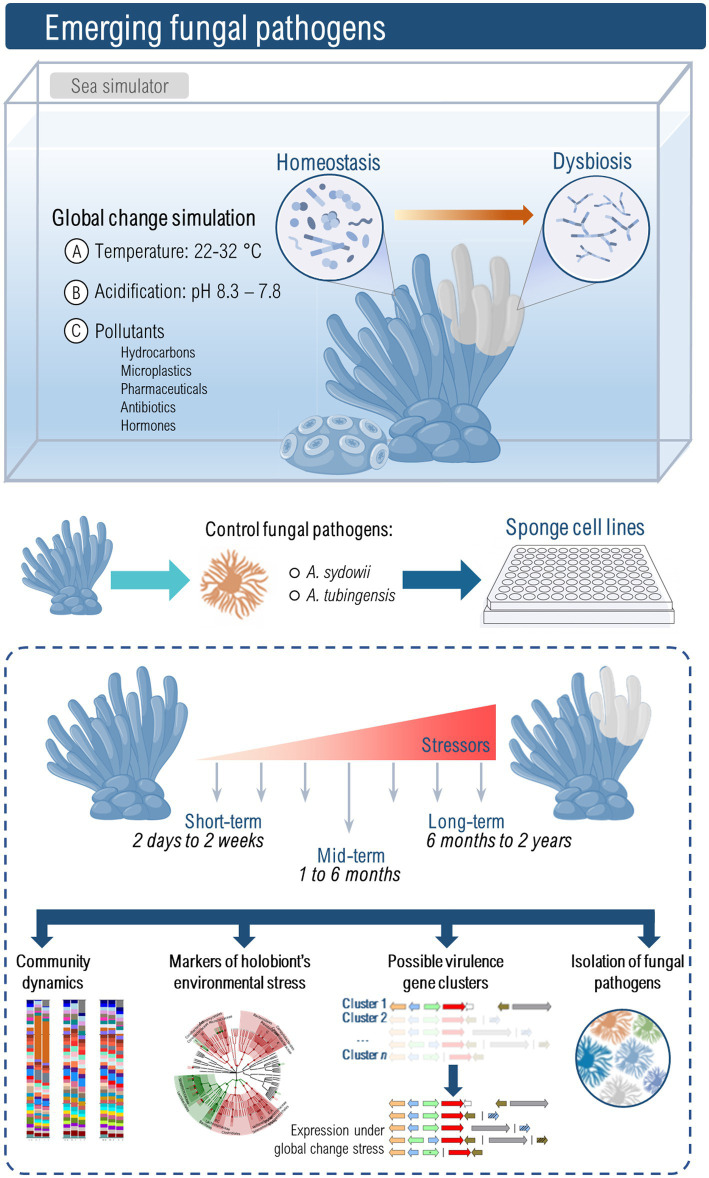
The study of emerging marine fungal pathogens due to global change requires dedicated facilities that perform mesocosm-style experiments, also known as sea simulators. Only a select few places worldwide have the characteristics of these kinds of laboratories, and therefore not many studies have been conducted in these settings (Krause et al., 2013b; Rosado et al., 2019; Luter et al., 2020). The variables controlled in these experiments include seawater temperature and pH, dissolved carbon dioxide, lighting, salinity, and the presence of pollutants such as hydrocarbons, microplastics, pharmaceuticals, hormones, or antibiotics. Mesocosm experiments allow an understanding of the multiscale temporal dynamics of gene expression, physiological traits, microbial community composition, and multispecies ecological interactions. These types of experiments are pivotal to identifying markers of holobiont stress or dysbiosis, changes in the immune status of the host in response to environmental conditions, activation of genes and clusters that promote fungal virulence, and the emergence of pathogens from previously innocuous strains. *Aspergillus tubingensis* or *A. sydowii* might become model fungal pathogens to understand fungal impact on resident microbial communities and immune responses of sponges. Naturally, this will require extreme measures of biosafety and containment since, in addition to infect —at least— one marine sponge species (Gautier et al., 2016), *A. tubingensis* is also a Risk Group 2 pathogen.

To advance our understanding of emerging fungal pathogens, it is crucial to implement novel experimental approaches (Figure 1). Until recently, the lack of sponge cell lines and cultures hindered our understanding of pathogenesis and sponge cellular response to infection. However, recent advancements have demonstrated the successful cultivation of sponge cells *in vitro* (Conkling et al., 2019; Hesp et al., 2023) and 3D cultures that mimic the architecture of sponge tissues (Urban-Gedamke et al., 2021), which should pave the way to more reproducible and mechanistic studies on host-pathogen interactions. The identification of fungi and other microbes that could shift away from mutualism/commensalism to pathogenicity requires the use of mesocosm experiments conducted under controlled conditions using marine simulator systems. These simulators will enable the emulation of different global change scenarios (e.g., light, temperature, CO_2_, salinity, pollutants) and facilitate long-term simulations, allowing for multi-generational studies. This approach would allow the identification of fungal taxa that could be harmful to marine sponges at different temporal scales. Moreover, it may also provide useful insights into fungal signatures, whether in the form of community structure or potential functionality, that could serve as predictors of sponge health within marine ecosystems.

### Fungal-based probiotics as sponge health promoters

In recent years, the potential use of microbial transplants and probiotics for treating a wide variety of human and animal disorders have emerged as novel paradigms (Peixoto et al., 2017, 2021; Rosado et al., 2019; Ushijima et al., 2023). Microbiome engineering offers a unique opportunity to improve not only human health but also agricultural productivity and climate management (Peixoto et al., 2017). However, environmental applications of microbiome engineering are progressing slowly because, even at a laboratory scale, they entail several logistical problems. Although some successful microbiome engineering applications have been reported (i.e., bioremediation, wastewater engineering, and biocontrol in agriculture), selecting appropriate strains, ensuring their sufficient abundance in the ecological unit to sustain a physiological benefit, and preventing contamination of non-target ecosystems, are still major concerns. For example, the deliberate release of inoculants, particularly in aquatic environments, could be detrimental to the resident microbial communities and to ecosystem functioning, as the dispersal of microbes is easier in aqueous media (Peixoto et al., 2017).

The use of microbial transplant and microbiome engineering in sponges has not been explored as in corals, the cnidarian animals that coincide with sponges in benthic ecosystems (Peixoto et al., 2017, 2021; Rosado et al., 2019). Although the use of probiotics in corals is in its infancy, several studies have been conducted to demonstrate the positive effect of bacterial inoculants on different coral species (Rosado et al., 2019). For example, some native bacterial populations enhance coral tolerance to a variety of pressures associated with global change (e.g., temperature stress or oil pollution) and are critical for nutrient cycling, coral defense responses, and coral health (Ziegler et al., 2019). Microbiota transplantation experiments, reported in the same study, have shown that these bacterial communities also promote the environmental plasticity of both coral and their associated microbiota when they are relocated to new habitats (Ziegler et al., 2019). In another example, Rosado et al. reported that manipulating a bacterial consortium of *Pseudoalteromonas* sp., *Holomonas taeanensis*, and *Cobetia marina*-related species, isolated from the coral *Pocillopora damicornis*, partially mitigated coral-bleaching and significantly improved the coral resistance to water warming. In addition, inoculation with this consortium controlled the development of the temperature-dependent pathogen *Vibrio coralliilyticus* (Rosado et al., 2019). However, the aforementioned development of coral probiotics has focused exclusively on bacteria, and no research has been conducted to date to investigate the use of fungi for enhancing health and stress tolerance in corals and sponges.

While fungi are not typically regarded as permanent members of the sponge holobiont, the use of fungal inoculants as probiotics presents an intriguing approach to transiently modulate microbial dynamics within the sponge, thereby aiding in the restoration of host physiology in the face of changing environmental conditions. In theory, by introducing fungal probiotics, the holobiont can undergo a transformative process, attaining a new stable configuration that is facilitated by the beneficial effects of these fungi. Once the desired recovery is achieved, and the use of the inoculant is discontinued, the holobiont would maintain this improved state.

Fungal probiotics have been used extensively as feed additive in fish, pigs, ruminants, poultry, and other animals. Yeasts, particularly those belonging to the *Saccharomyces* genus, have been studied for their ability to positively influence the gut microbiota and overall digestive processes. In humans, *S. boulardii* has shown health-promoting effects and is commonly used to treat multiple gastrointestinal diseases (Pais et al., 2020; Abid et al., 2022). In fish, the yeasts *S. cerevisiae*, *Geotrichum candidum*, and *Yarrowia lipolytica* have been investigated for their probiotic potential, both alone and in combination with bacterial strains (Melo-Bolívar et al., 2021; Reyes-Becerril et al., 2021). Filamentous fungi, such as *Aspergillus niger* or *A. oryzae*, have also been used in fish to improve digestion, nutrient utilization, and immunity (Dawood et al., 2020; Shukry et al., 2021; Jasim et al., 2022). These findings suggest that both unicellular and filamentous fungi play significant roles in modulating host physiology and highlight their potential to perform similar functions in sponges.

The factors required in a workflow to isolate and characterize novel candidates for coral probiotics have been previously highlighted and seem to apply to sponges as well. Examples of desirable roles for these probiotic strains could be the supply of nutrients and their cycling, modulation of immune responses, UV protection, dissemination of beneficial genes/pathways, and the biological control of pathogen populations, among others (Peixoto et al., 2021).

**Figure 2:**
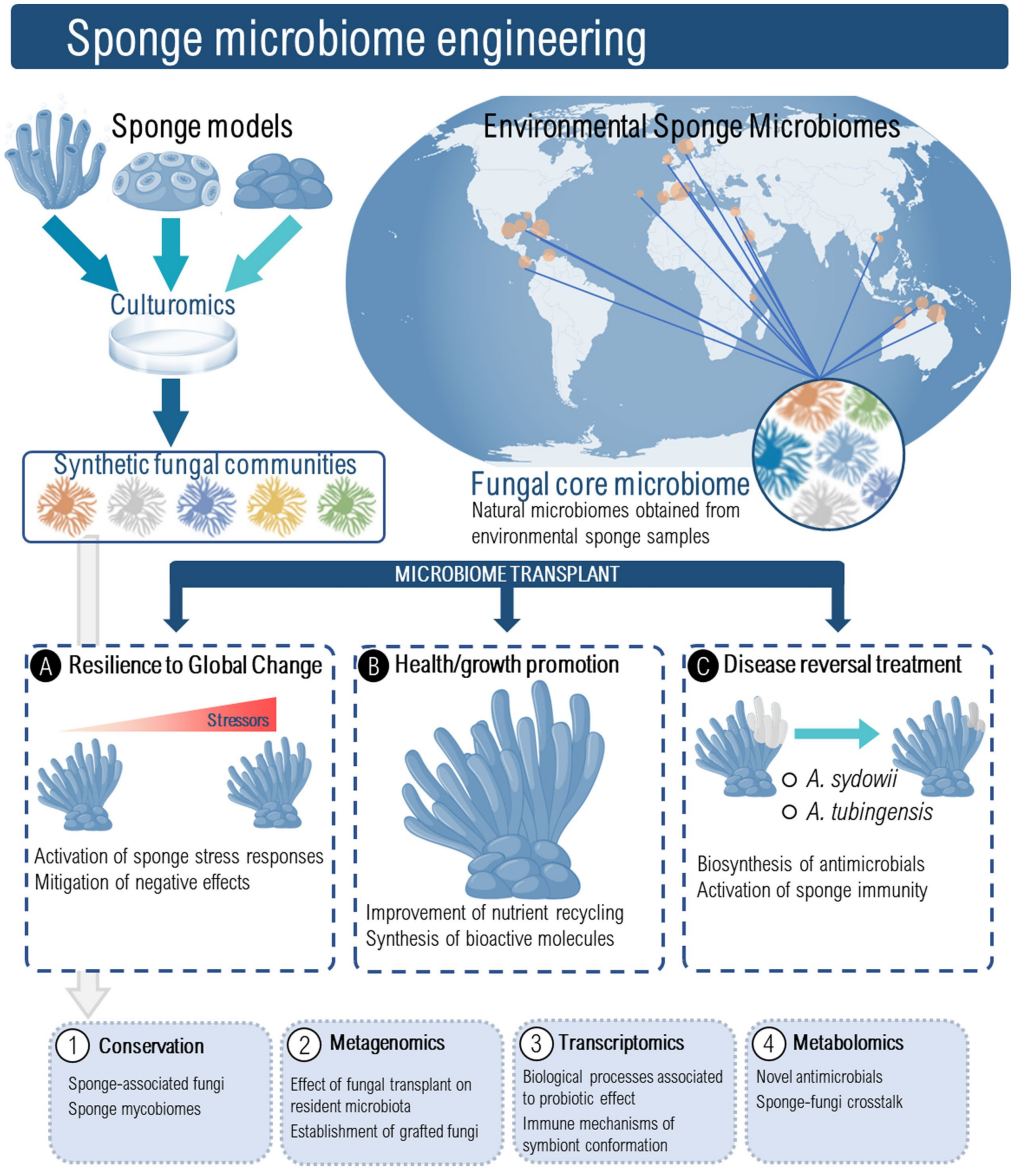
Sponge microbiome engineering using sponge associated fungi could serve as a novel strategy to induce resilience to global change, promote sponge health and growth, and as treatment for disease reversal. Fungal probiotics might be prospected from laboratory and aquaculture sponge models as well as from environmental sponge mycobiomes. The conservation and characterization of these fungal probiotics should encompass a multi-omics approach to understand the effect of fungal transplant on resident microbiota and its persistence, the immune mechanisms of holobiont confirmation, and the molecular crosstalk between sponges and their resident microbes under global change stress conditions.

Manipulating sponge-associated fungal communities may be an important new strategy to cope with the threats posed to sponge health by pathogens and pollutants (Figure 2). Additionally, we anticipate that sponge-derived fungi might be used as novel sponge health promoters, to increase the sponge’s resistance to opportunistic infections under a scenario of global change. To develop fungal-based probiotics for marine sponges several hurdles should be overcome, and crucial questions must be first elucidated. These include (but are not limited to): (1) What roles do fungi play in sponge ecosystems? (2) How can fungi influence sponge immune functions? (3) Which fungi are essential for sponge ecosystem functioning? (4) How do the fungal communities interact with other members of the sponge microbiome? (5) How do sponge-associated fungi respond to environmental perturbations? (6) What are the critical steps in isolating and selecting putative beneficial sponge microbes? and (7) What is the best way for inoculating fungi in sponge ecosystems? In addition, pilot-scale trials will need to be undertaken involving the use of fungal-based probiotics, under controlled aquarium conditions to prove that the overall concept of sponge probiotics is in fact viable (Figure 2).

### Concluding remarks

Fungi are thought to be relevant sponge pathogens but there is scarce knowledge regarding the potential role of fungi as opportunistic —even emergent— pathogens in the field of sponge biology, particularly in the context of stress due to global change scenarios. In this setting, global change might cause microbial partners, particularly fungi, to flip from symbiosis to pathogenicity. We hypothesize that anthropogenic activities and climate change may reveal sponge-associated fungi as novel emerging pathogens. Global change scenarios could trigger the expression of fungal virulence genes and unearth new opportunistic pathogens, posing a risk to the health of sponges thus severely damaging reef ecosystems. Although it is not yet clear that such a scenario could come to pass, this hypothesis should be investigated to enable timely bioprospection and interventions. This knowledge could lead to identifying sponge-associated fungi as potential indicators or “forecasters” of environmental perturbations or as tools to promote sponge health under stress.

## Author contributions

The first draft of the manuscript was written by YP-L, LY, and RB-G. Figures were prepared by YP-L and RB-G. All authors commented on previous versions of the manuscript, read and approved the final manuscript.

## Funding

This project has received funding from the European Union’s Horizon 2020 research and innovation programme under Grant Agreement no. 101000392 (MARBLES). This output reflects only the author’s view, and the Research Executive Agency (REA) cannot be held responsible for any use that may be made of the information contained therein. YP-L received postdoctoral fellowships from UNAM-DGAPA and from CONACyT (CVU: 697462). RB-G received a Sabbatical fellowship (CVU: 389616) from the National Council of Science and Technology (CONACyT), Mexico.

## Conflict of interest

The authors declare that the research was conducted in the absence of any commercial or financial relationships that could be construed as a potential conflict of interest.

## Publisher’s note

All claims expressed in this article are solely those of the authors and do not necessarily represent those of their affiliated organizations, or those of the publisher, the editors and the reviewers. Any product that may be evaluated in this article, or claim that may be made by its manufacturer, is not guaranteed or endorsed by the publisher.
